# Analysis of Autoencoders for Network Intrusion Detection †

**DOI:** 10.3390/s21134294

**Published:** 2021-06-23

**Authors:** Youngrok Song, Sangwon Hyun, Yun-Gyung Cheong

**Affiliations:** 1 Department of AI, Sungkyunkwan University, Suwon 16419, Gyeonggi-do, Korea; id4thomas@g.skku.edu; 2 Department of Computer Engineering, Myongji University, Yongin 17058, Gyeonggi-do, Korea

**Keywords:** IDS, NIDS, ML-NIDS, autoencoders, deep-learning models, model design, unsupervised learning Algorithms, IoT

## Abstract

As network attacks are constantly and dramatically evolving, demonstrating new patterns, intelligent Network Intrusion Detection Systems (NIDS), using deep-learning techniques, have been actively studied to tackle these problems. Recently, various autoencoders have been used for NIDS in order to accurately and promptly detect unknown types of attacks (i.e., zero-day attacks) and also alleviate the burden of the laborious labeling task. Although the autoencoders are effective in detecting unknown types of attacks, it takes tremendous time and effort to find the optimal model architecture and hyperparameter settings of the autoencoders that result in the best detection performance. This can be an obstacle that hinders practical applications of autoencoder-based NIDS. To address this challenge, we rigorously study autoencoders using the benchmark datasets, NSL-KDD, IoTID20, and N-BaIoT. We evaluate multiple combinations of different model structures and latent sizes, using a simple autoencoder model. The results indicate that the latent size of an autoencoder model can have a significant impact on the IDS performance.

## 1. Introduction

It is critical to defend network systems and information assets from network attacks, and there exist various techniques to deal with network attacks. Among those, public key cryptography and digital certificates can be used to protect network systems by enabling source authentication. These cryptographic techniques make it possible to verify whether network traffic is originated from a trusted source or not. Thus, we can filter out malicious traffic from untrusted sources. With the advent of quantum computing, classical public key cryptography will face limitations in terms of security [1], thus post-quantum public key cryptography is being developed to replace them [2,3]. In addition, research on public key certificate for constructing public key infrastructure in post-quantum public key cryptosystems is also conducted [4]. Despite such cryptographic countermeasures, it may be possible for attackers to compromise and misuse legitimate hosts for her attacks. Unfortunately, in this attack scenario, source authentication based on public key cryptography and digital certificates has a limitation that it cannot filter attack traffic from the host that is cryptographically legitimate but compromised by the attacker. To address this limitation, network intrusion detection systems are widely used as an effective countermeasure against network attacks.

Network intrusion detection systems using machine-learning or deep-learning techniques (simply called ML-NIDS) have gained a lot of attention in intelligently monitoring network traffic for highly accurate detection [5,6]. ML-NIDS can be divided into two categories: the signature-based method and the anomaly-based method. The signature-based ML-NIDS method generally assumes that pre-labeled attack traffic data are made available in advance, and learns those known intrusion patterns using supervised learning algorithms [7,8], such as SVM [9,10] and Decision Tree [11]. Khan et al. [12] used a particle swarm optimization method to select the optimal feature subset from a given dataset in terms of detection accuracy. The authors then evaluated several machine-learning algorithms such as random forest and neural network as classifiers using KDD Cup 99 [13] and NSL-KDD [14] datasets. During the last decade, various deep-learning algorithms have been used for more accurately classifying network attacks. Salama et al. [15] leveraged a deep belief network (DBN) for dimensional reduction and applied SVM to detect the attack in the NSL-KDD data. Kim et al. [16] built a NIDS using LSTM (Long Short-Term Memory) networks. Alom et al. [17] proposed a DBN composed of stacked Restricted Boltzmann Machines (RBMs). This approach was evaluated with the NSL-KDD data set, and achieved an accuracy of 97.5%.

However, with the continuous development of new attack techniques, network attacks keep evolving into new patterns [18], and this makes it infeasible to predict previously unseen attack patterns. Due to unpredictable attack fashions, traditional signature-based NIDS, relying only on previously known attack patterns, encountered limitations in detecting unknown and sophisticated attacks in real time [19]. Moreover, signature-based methods rely on pre-labeled train data, but the labeling process generally needs tremendous human efforts.

These limitations were addressed by the anomaly-based approach, which enables us to quickly detect and respond to unknown attack patterns to stabilize network operation while reducing human intervention [20,21,22,23,24]. The anomaly-based approach generally learns normal patterns in an unsupervised manner and detects anomaly samples whose patterns significantly deviate from the normal ones. For instance, the one-class SVM method regards the majority of the data located densely within a specific area as normal and a few outliers as abnormal. One-class SVMs [23] and OptiGrid clustering-based methods [24] were used to detect attack traffics.

**Figure 1:**
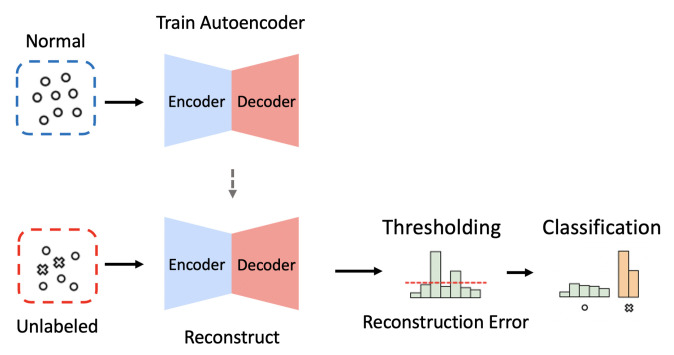
The overall process of intrusion detection when using the dimension reduction (i.e., Encoder) and reconstruction (i.e., Decoder) methods of autoencoders.

Several groups of researchers recently developed NIDS using *autoencoders*, a generative deep-learning model consisting of an encoder and a decoder [25,26,27]. Given an input of *M*-dimensional vector, the encoder compresses it into a latent vector represented as a *N*-dimensional vector where M>N, and the decoder subsequently reconstructs the compressed latent vector back to the *M*-dimensional vector (see Figure 1). An autoencoder-based NIDS trained with only normal traffic data is expected to recover any given input as close as possible to the learned normal patterns. Therefore, we can classify an input instance as an attack if its reconstruction error is larger than a predefined threshold; otherwise, we can classify the input instance as normal. In this fashion, an autoencoder-based NIDS is capable of detecting unknown types of attacks when their patterns deviate from the learned normal patterns.

Despite the effectiveness of autoencoders in detecting unknown types of attacks, the detection performance of an autoencoder-based NIDS can be very sensitive to the architecture and hyperparameter settings of the underlying autoencoder model. Thus, it is critical to find the optimal configurations of autoencoders that can reach the best detection performance. Most of the previous papers have reported manually obtained best models by conducting numerous experiments with particular data sets. Such manual processes generally take tremendous time and effort and also have to be repeated as data changes. This may result in considerable delays in the development of an autoencoder-based NIDS and consequently degrades the practical applicability of autoencoders.

When implementing an autoencoder-based NIDS, it is required to make decisions related to the following model design issues:

- How many hidden layers and how many neurons for each layer are necessary? Is a bigger model better for intrusion detection?
- What dimension of the latent layer is appropriate to represent the data? For instance, the model may not represent the data well if its latent layer has only one neuron. In that case, the latent layer is likely to be too generic to characterize the data. On the other hand, the model would not benefit the advantage of generalization that occurs during the compression/reconstruction process if its latent layer contains an excessive number of neurons.
- How do we set a threshold that divides the normal and abnormal data, when labeled data are not available?
- What metrics are best to represent the difference between the input and its output (e.g., L1, L2, Cross-Entropy)?

To our knowledge, these critical engineering issues have been little studied.

This paper makes three contributions. First, we present the engineering issues in the application of autoencoders for anomaly-based NIDS. Second, we investigate how the model size and the latent size of autoencoders have an impact on the intrusion detection performance. Specifically, we design and perform experiments to test the efficacy of some of the aforementioned factors using benchmark datasets that contain attack traffic data collected from either conventional or IoT networks. Finally, we analyze the experiment results and report the findings.

This paper is organized as follows. Section 2 reviews the related work. We describe the data used for our study in Section 3 and the evaluation methods in Section 4. Section 5 presents our experiment results followed by discussions. We conclude with a summary and suggestions for future work.

## 2. Related Work

This section reviews the previous works in ML-NIDS. First, we describe traditional works that use deep-learning and recent works that employ ensemble learning which can boost the IDS performance when combined with deep-learning algorithms. Second, we look into autoencoders for feature extraction to enhance the classification performance. Third, we review autoencoder-based network intrusion detection systems.

### 2.1. Intrusion Detection with Deep-Learning and Ensemble Learning

There exists literature on network intrusion detection using deep-learning techniques, such as restricted Boltzmann machine (RBM), deep Boltzmann machine (DBM), deep belief network (DBN), and deep neural network (DNN). Kim et al. [16] proposed an IDS based on recurrent neural networks (RNN), where a LSTM (long short-term memory) architecture was applied to the RNN. Tang et al. [28] designed an IDS based on deep neural networks (DNN) for flow-based anomaly detection in software-defined networking environments.

Several intrusion detection methods based on ensemble learning and deep-learning are presented. Zhang et al. [29] proposed ML-NIDS based on multi-dimensional feature fusion and an ensemble learning approach. This method extracts several basic feature datasets from raw data and then constructs multiple comprehensive feature datasets by combining the basic feature datasets. The comprehensive feature datasets are used to train different base classifiers constituting the ensemble learning model. Yong et al. [30] studied ensemble learning approaches to detecting the webshell attack in IoT networks. Specifically, the authors compared the effectiveness of ensemble machine-learning approaches under several different settings: an ensemble of either homogeneous or heterogeneous classification models. Hemalatha et al. [31] proposed a malware detection method based on deep learning. This method transforms program binary files into two-dimensional images and used a pretrained deep-learning model called DenseNet to classify the binary images. The authors additionally addressed the class imbalance issue typically shown in malware datasets by employing the reweighted cross-entropy loss function.

### 2.2. Autoencoders for Feature Reduction

Diverse autoencoder models have been leveraged as feature learning methods and combined with supervised learning algorithms to detect attack classes. For instance, Li et al. [32] presented an ML-NIDS that uses an autoencoder for dimension reduction and a DBN for classification. The DBN consists of multiple layers of RBMs and an additional layer of BP neural network as a classifier which distinguishes malicious samples from normal samples. Their system was evaluated on the KDD Cup 99 dataset, and the results showed that combining an autoencoder and DBN can achieve better detection accuracy than working with DBN alone. Tao et al. [33] proposed a data fusion approach based on the Fisher score and deep autoencoder to reduce the dimensionality of data. Using the KDD Cup 99 dataset, it was confirmed that integrating the deep autoencoder as a feature extraction method can improve the accuracy of classification algorithms such as J48, the backpropagation neural network, and SVM.

Although the KDD Cup 99 dataset was popularly used to evaluate NIDS in 2000s, it has shortcomings of contains duplicate records in training and test sets, making it inadequate for benchmark data [8]. Hence, the NSL-KDD data were created by removing redundancies in the training and test sets of the KDD set [14]. Javaid et al. [34] developed an ML-NIDS using a combination of sparse autoencoder and SoftMax regression (SMR) method. Through evaluations using the NSL-KDD dataset, the authors confirmed that using an autoencoder as a feature extraction method can improve NIDS performance. Shone et al. [35] proposed a non-symmetric deep autoencoder (NDAE) which provides unsupervised feature learning and data dimensionality reduction. They developed an ML-NIDS that combines stacked NDAEs and Random Forest (RF), achieving 97.85% and 85.42% accuracy for the KDD Cup 99 and NSL-KDD dataset, respectively. This indicates that the data duplication in the KDD Cup 99 facilitates the anomaly detection task. Al-Qatf et al. [36] combined sparse autoencoders and SVM, where the autoencoders perform feature learning and dimensionality reduction and SVM classifies the data as normal or attack. This approach was evaluated on the NSL-KDD dataset and obtained an F1-score of 85.28%.

Madani et al. [37] evaluated the robustness of autoencoder models against intentional data poisoning. They analyzed and compared the performance of an autoencoder-based model and a PCA-based model under adversarial contamination attacks using the NSL-KDD dataset. The results showed that the autoencoder-based NIDS outperformed the PCA-based NIDS approximately 15% in detection rate.

Yu et al. [38] used a stack of dilated convolutional autoencoders to extract important features from a large volume of unlabeled traffic data in the CTU-UNB and Contagio-CTU-UNB datasets which contain normal and botnet network traffic data. Thing et al. [39] also used stacked autoencoders to learn important features for the IEEE 802.11 wireless network attack classification. Zhang et al. [40] applied a similar architecture which consists of sparse stacked autoencoders and binary tree ensemble method. When evaluated on the NSL-KDD dataset, their approach achieved an F1-score of 91.97%.

In IoT environments, Dutta et al. [41] developed an anomaly detection system using a deep sparse autoencoder, a DNN, and a LSTM followed by a logistic regression classifier which detects anomalous traffic. The deep sparse autoencoder compresses the input data to alleviate the computational cost in the classifier. Several network traffic datasets (e.g., IoT-23 [42], LITNET-2020 [43] and NetML-2020 [44]) collected in IoT environments were used to evaluate their system.

### 2.3. Autoencoders for Anomaly Detection

This section reviews the application of autoencoder models that detect network intrusions in an unsupervised manner, which is closely related to our approach. Lopez-Martin et al. [45] proposed a classification model using conditional variational autoencoders to detect and classify the five types of labels (normal, DoS, R2L, Probe, and U2R) in the NSL-KDD data. In particular, given a labeled training dataset, the decoding network of the proposed model is additionally trained with the class labels associated with the trained data logs. When reconstructing from a compressed vector, the decoding network predicts the class label associated with the reconstructed data features. Thus, this approach requires pre-labeled data for training, unlike our unsupervised-based approach. The task of classifying normal labels against the other four labels achieved an F1-score of 0.83.

Aygun et al. [26] compared two different autoencoder models for detecting unknown types of attacks. An autoencoder and a denoising autoencoder were evaluated with NSL-KDD dataset, and achieved the F1 of 89.51% and 89.28% respectively. To classify a given data as either normal or abnormal, they stochastically select a threshold, and this supplements our own study. Mirsky et al. [25] employed an ensemble scheme to develop an ML-NIDS named Kitsune. The proposed model first maps the features of a given network packet into an ensemble of autoencoders. Then each autoencoder reconstructs the packet features and computes root mean squared error (RMSE) as the reconstruction error. Finally, the model summarizes the RSMEs from each autoencoder and checks whether the final result exceeds a predefined threshold. In particular, they studied how to distribute the entire set of data features to each autoencoder and how to derive the final decision by collecting and analyzing the results from each authencoder. Their work was applied to online intrusion data which are not publicly accessible for comparative evaluation.

These autoencoder-based methods are applied to build NIDS in IoT environments recently. Meidan et al. [46] used deep autoencoders to detect IoT botnet attacks. The proposed model is composed of a set of autoencoders, each autoencoder learns the normal network behaviors of the designated IoT device and detects anomalous traffic from the device.The proposed model was evaluated on a testbed network consisting of nine commercial IoT devices infected by Mirai and BASHLITE botnets. The model achieved a true positive rate of 100% and a false positive rate of 7%.

Similarly, Shahid et al. [47] presented an ML-NIDS to detect anomalous traffic in IoT networks. To consider device diversity commonly assumed in IoT networks, the proposed system consists of multiple sparse autoencoders, each dedicated to one designated device type in a target network environment to be monitored. Each sparse autoencoder is trained to learn the normal communication pattern of the corresponding type of device. When new network traffic is observed, every autoencoder in the system first compresses and then reconstructs incoming data as close to the original traffic data as possible. If at least one of the autoencoders successfully reconstructs the given traffic data, the system considers it as legitimate; otherwise, if all of the autoencoders fail to reconstruct the given traffic data, it is considered as anomalous traffic. The authors tested the proposed system with the dataset collected from IoTPOT [48], and the proposed system reported true positive rate of 86.9% to 91.2% depending on a parameter setting, and its false positive rate was from 0.1% to 0.5%.

As described above, diverse autoencoder models and their ensemble are used for detecting network intrusions in an unsupervised manner and report promising results in various evaluation metrics. However, it was not clearly disclosed how a particular model architecture and its hyperparameters were designed. Unfortunately, the importance of model configuration is largely overlooked in the previous works. In this article, we evaluate how the size and the latent dimension of autoencoders influence the NIDS performance, to tackle the practical issues explained above.

## 3. Datasets

Several labeled network traffic datasets are available for evaluating intrusion detection systems. We selected three datasets for our experiments, NSL-KDD, IoTID20, and N-BaIoT. NSL-KDD [14] dataset was created by removing redundancy from training and test sets of the KDD Cup 99 dataset [13], which is the most widely known dataset for measuring IDS performance. We also employed two IoT datasets, IoTID20 [49] and N-BaIoT [46], which are up to date and were collected using real devices in IoT environments.

### 3.1. NSL-KDD Data

The original NSL-KDD data consists of training data and test data. To create a validation set, we randomly sampled 10% from the NSL-KDD training data and use them as our validation set. In NSL-KDD, there are four attack categories provided in the dataset; DoS (denial of service), U2R(unauthorized access to local root privileges), R2L (unauthorized access from remote machine), and Probe (surveillance and other scanning). We categorized the attack types present in the dataset into four categories as set out in Yang et al. [50]. Table 1 presents the number of samples from each set and attack category.

In the preprocessing step, we convert non-numerical data into numerical data to build an input for the autoencoder. *Protocol, Service* and *Flag* features are treated as categorical data. *Protocol* feature denotes a network protocol whose value can be either *tcp, udp*, or *icmp*. When using one-hot encoding, the *Protocol* feature is represented as a 3-dimensional vector. The *Service* feature contains 86 distinct values, and thus is represented as an 86-dimensional vector. *Flag* has 13 different values and is represented as a 13-dimensional vector. The *Source_port_number* and *Destination_port_number* range from 0 to 65,535, and we represent it as a 3-vector, dividing the number into well-known port (0–1023), registered port (1024–49,151), and dynamic port (49,152–65,536).

For numeric data, we normalize the numerical values between 0 and 1. The *Duration, Source_bytes, and Destination_bytes* features and the features suffixed with *count* (e.g., *Dst_host_count*, *Dst_host_srv_count*) are normalized using the min-max scaling, after statistical outliers are eliminated. The feature values suffixed with *rate* (e.g., *Serror_rate, Dst_host_serror_rate*) are between 0 and 1, and thus are used as-is, without conversion. Finally, the data are represented as 114-dimensional vectors by combining 12 general features and 112 one-hot encoded categorical features.

### 3.2. IoTID20 Data

IoTID20 data were collected using two victim IoT devices connected to a Wi-Fi router of a smart home network environment [49]. Each sample consists of 83 network features and three label features. We randomly split the entire data into training, validation and test sets at a ratio of 8:1:1. Table 2 shows the number of samples in each set and category.

In the preprocessing step, we first remove samples containing the *NaN* and *INF* feature values. We also eliminate the *Timestamp, FlowID, SrcIP, and DstIP* features to prevent the model from learning specific logs during validation. Then we scale the numerical features using a min-max scaler. The three categorical features *Src Port, Dst Port, and Protocol* are respectively processed into a categorical one-hot vector. The *Port* values are divided into three categories. We convert the *Protocol* feature, one of three values (0,6,17), as a three-dimensional one-hot vector. The resulting processed data have 77 numerical features and 9 categorical features.

### 3.3. N-BaIoT Data

N-BaIoT data consists of the traffic data collected from nine commercial IoT devices before and after they are infected by Mirai and BASHLITE botnets. Each data sample consists of 23 features of traffic statistics such as packet count, the size of inbound and outbound packets, and the inter-arrival times of packets for five time windows.

The dataset contains the samples collected from the nine devices during 10 types of attacks executed by Mirai and BASHLITE. We selected the data samples from five out of the nine devices for our evaluation: Danmini Doorbell, Ecobee Thermostat, Provision PT-737E, Philips B120N/10 Baby Monitor, and SimpleHome XCS7-1002-WHT. The data samples of each device are divided into training, validation, and test set at a ratio of 8:1:1, respectively. Table 3 shows the number of samples in each set and category.

Numerical features were scaled using a min-max scaler into 116 features. Preprocessing was done separately for each device.

## 4. Approach

This section describes our experiment approach to studying the impact of the deep-learning model design on the performance of intrusion detection. Specifically, we test how the autoencoder model structure and the size of its latent dimension affect the ML-NIDS performance.

### 4.1. Overview

**Figure 2:**
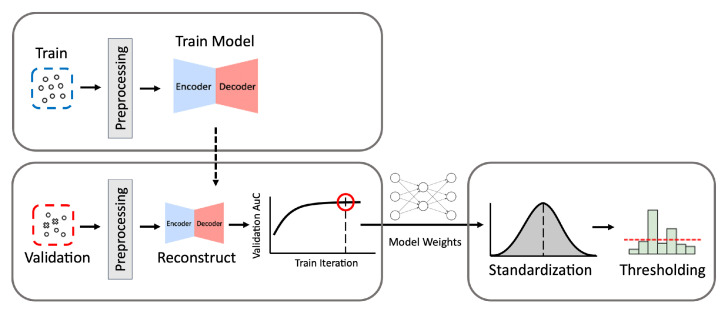
The process of building autoencoder models. *Train* denotes the data for training, and *Validation* denotes the data for validation. The process consists of three phases: training, model selection, and validation.

Our process to build the optimal autoencoder model is described below and in Figure 2.

(1) Preprocessing: Given a training data set, we first remove attack samples from the training set so that only normal samples are used to train the autoencoder. Then we vectorize the data by converting the feature values into numerical vectors using the min-max normalization. The features are scaled using the minimum and maximum values obtained from the training set. Additional preprocessing methods applied to each particular data set are described in detail in Section 3.
(2) Training: The autoencoder model is trained to reconstruct any given input as close as possible to the normal log patterns of the training set and L2 norm is used as reconstruction loss. Theoretically, the autoencoder model trained with normal logs is expected to recover any input log as close as possible to the learned normal log patterns. Due to this, if a given input in fact belongs to an attack class, it is likely that the recovered output would be significantly different from the original input. This means that the class of an input sample is determined based on the difference between the input and its output; if the difference is greater than a set threshold, it is classified as an attack. Otherwise, it is classified as normal.
(3) Model Selection: At each epoch during the training phase, the Area under the Curve (AuC) score of the Receiver Operating Characteristic (ROC) curve is calculated using the validation data which contain the normal and attack logs. The AuC score enables us to measure the model’s overall performance as the model weights change. During the training phase, we record the highest AuC score and its corresponding model weights to obtain the best-performing model. When the training phase is completed, we use the model weights that resulted in the highest AuC score.
(4) Threshold Selection: Since the threshold on the reconstruction distance between an input and its output divides the normal and abnormal (i.e., attack in this study) classes, selecting its value has a tremendous impact on the ML-NIDS performance. We evaluate the model’s performance using the validation set, varying the threshold value to determine the optimal figure for the trained model. We use the Z-score of standard normal distribution as the threshold metric. Using the Z-score instead of the reconstruction error values before normalization can facilitate the reproduction of the classifier even when reconstruction error values in unnormalized form change depending on a data set.
  To set the threshold Z-score, we first standardize reconstruction distances with the mean and variance values of reconstruction distances calculated using only the normal samples in the validation data. To find the best-performing threshold, we inspect the range of (−4,4) with 0.01 interval, which covers 99.994% of the standard normal distribution. We determine the Z-score value that best divides the normal and abnormal classes in the validation data as the threshold Th.
(5) Evaluation: Finally, we evaluate the ML-NIDS performance using the test data. We compute the Z-score of each log’s reconstruction error using the mean and variance of normal samples in the validation set calculated in Step 4. We classify each log as an attack if its Z-score exceeds Th. Otherwise, it is classified as normal. By comparing these predicted labels and the test data’s original labels, we calculate the model’s evaluation metrics (i.e., accuracy, TPR, FPR, and MCC explained in Section 4.3).

### 4.2. Model Design

As Section 2 summarizes, various autoencoder models are used as (one-class) unsupervised machine-learning algorithms for anomaly-based intrusion detection systems. We experiment with different model configurations to test whether an autoencoder model’s design choices influence intrusion detection performance.

**Figure 3:**
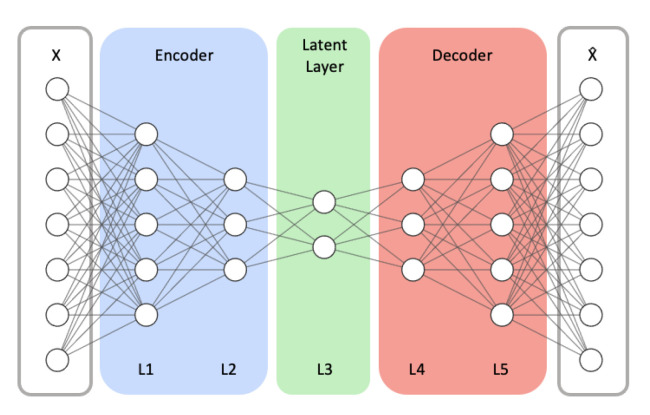
An example autoencoder model architecture with symmetrical encoder and decoder networks. *X* and $\hat{X}$ respectively represent the model’s input and its reconstructed output.

In this study, we focus on two essential design aspects of autoencoder models: *model structure* and *latent size*. *Model Structure* represents the number of hidden layers (depth) and the number of neurons (width) in each hidden layer of the encoder and decoder networks. We vary the model’s depth and width to inspect how the model’s capacity and complexity affect the detection performance. In our study, *Latent size* denotes the dimension of the latent layer, which is illustrated as the L3 layer in Figure 3.

Autoencoder models can be regarded as a dimension reduction algorithm trained to compress their input into a latent representation. Therefore, if the size of the latent layer is too small, the model may not represent the data well because the latent vector is too generic to characterize the data. On the other hand, a model would not benefit from generalization during the compression/reconstruction process if its latent layer contains an excessive number of nodes. Thus, we also test different latent sizes for each *Model Structure* to assess its impact.

### 4.3. The Evaluation Metrics

For evaluation, we report the standard metrics such as accuracy, true positive rate, and false positive rate for comparison between different model configurations. True Positive Rate (TPR) records the attack detection rate. False Positive Rate (FPR), also known as false-alarm rate, is an important measure for evaluating an IDS, since an anomaly-based IDS tends to yield high false-alarm rate [51]. TPR, FPR, Accuracy, and F1 are calculated by the following equations where *TP* denotes true positives, *TN* denotes true negatives, *FP* denotes false positives, and *FN* denotes false negatives.

(1) $$\mathrm{True}\;\mathrm{Positive}\;\mathrm{Rate}\;\left(\mathrm{TPR}\right)\;=\frac{TP}{TP+FN}$$

(2) $$\mathrm{False}\;\mathrm{Positive}\;\mathrm{Rate}\;\left(\mathrm{FPR}\right)\;=\frac{FP}{FP+TN}$$

(3) $$\mathrm{Accuracy}\;=\frac{TP+TN}{TP+FP+TN+FN}$$

(4) $$F1\;=\frac{TP}{TP+\frac{1}{2}\left(FP+FN\right)}$$

Furthermore, we use Matthews Correlation Coefficient (MCC) as the primary metric to represent the best performance that the model can achieve when the threshold is fixed. The score is computed using Equation (5) and is in the range of (−1,1), where +1 indicates a perfect prediction and −1 means that all predictions are wrong.

(5) $$\mathrm{MCC}\;=\frac{TP\cdot TN-FP\cdot FN}{\sqrt{\left(TP+FP)\cdot (TP+FN)\cdot (TN+FP)\cdot (TN+FN\right)}}$$

MCC is considered a balanced measure because a high score can be obtained when the prediction results are good across all the classes and not just in one particular class. Hence, MCC can effectively represent the model’s performance even when there is class imbalance [52] and is used for measuring IDS classification performance [41]. Most benchmark IDS datasets are imbalanced, with more attacks than normal logs, since the data are usually collected via simulations.

## 5. Results and Analysis

This section describes the evaluations we carried out to study how the autoencoder model’s design (i.e., model structure and latent size) influences the NIDS performance with a combination of different model configurations. We report the average performance of multiple runs on the given test set using different random seeds due to the performance variance between runs. The random seeds are associated with random batch sampling of training data and model weight initialization.

### 5.1. Model Configurations

We test multiple combinations of different *Model Structures* and *Latent Sizes* using an autoencoder model, being comprised of fully connected hidden layers with the ReLU activation function and an output layer with the Sigmoid activation function. For evaluation, we tested three *Model Structure* configurations. We represent each Model Structure configuration as the form of **(Depth**, **Size**). **Depth** denotes the number of fully connected hidden layers of the model. **Size** represents the number of nodes of the encoder’s first hidden layer (L1 in Figure 3). The next hidden layer in the encoder is half the size of its previous hidden layer. In this fashion, the size of each hidden layer in the encoder is reduced by half down to the middle layer (i.e., L3 in Figure 3), representing the compressed vector. The decoder is symmetric to the encoder.

The three model configurations used in our evaluation are structured as follows in Table 4.

Using these configurations, we test if the model capacity affects IDS performance by comparing the (**5**,**32**) model with the (**5**,**64**) model, since they have the same number of hidden layers but differ in the number of trainable parameters. To analyze the impact of the model complexity, we compare the (**5**,**64**) model with the (**7**,**64**) model because they have similar number of trainable parameters and different number of hidden layers.

For each *Model Structure*, we inspect the potential latent sizes, ranging from 1 up to (the size of the hidden layer adjacent to the latent layer) −1. For example, in the (**5**,**32**) model and the (**7**,**64**) model, we vary the latent layer dimensions ranging from 1 to 15. For the (**5**,**64**) model, we test the latent sizes of 1 to 31. These models are trained to minimize the reconstruction loss using the Adam optimizer [53], at the learning rate of 1 × 10${}^{-4}$, and the weight decay value of 1 × 10${}^{-5}$. No other regularization methods were used.

### 5.2. Results: NSL-KDD Data

This section presents the results of our experiments when the NSL-KDD data are employed. We report the average of 20 test runs. In each run, a model is trained for 100 epochs with the batch size of 512. We obtained the best MCC of 0.712 when using the (**5**,**64**) model with the latent size of 3, as displayed in Table 5. We chose the best-performing latent size based on the MCC score for each model structure and present its performance scores along with its latent size and threshold.

In terms of *Model Structures*, the (**5**,**64**) and (**7**,**64**) models generally outperform the (**5**,**32**) model, except for the FPR. This suggests that the model capacity has an impact on the IDS performance, as the (**5**,**64**) model has about twice the number of trainable parameters of 18,880 compared with the (**5**,**32**) model’s 8,448 trainable parameters. When the model capacities are similar, as in the (**5**,**64**) and (**7**,**64**) models, the impact of the number of hidden layers on the detection performance is marginal.

Table 6 reports the best performance among the 20 runs for each model configuration. The TPR, FPR, accuracy, MCC, and F1-scores are presented. The best F1-score is 0.895 using the (**5**,**32**) model and the latent size of 4. This is comparable to the best F1-score of 0.895 reported in [26]. Table 7 displays the confusion matrix of the best-performing model for each configuration. The models tend to predict attacks as normal logs more often than to predict normal logs as attack.

**Figure 4:**
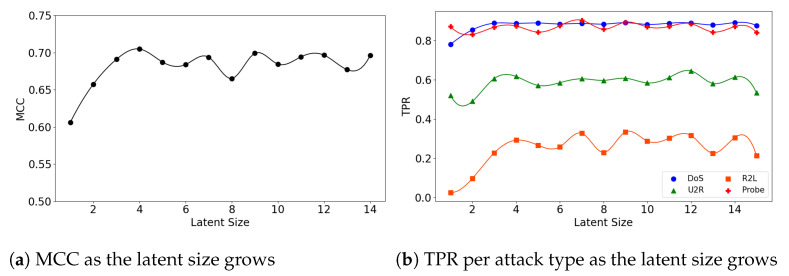
The results of evaluation using the NSL-KDD dataset when the (**5**,**32**) model is employed.

**Figure 5:**
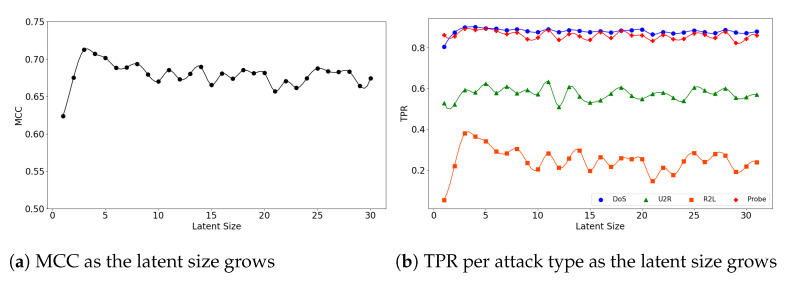
The results of evaluation using the NSL-KDD dataset when the (**5**,**64**) model is employed.

**Figure 6:**
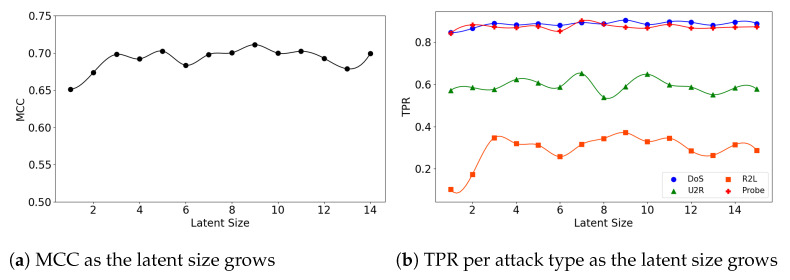
The results of evaluation using the NSL-KDD dataset when the (**7**,**64**) model is employed.

In the second experiment, we look at the interplay of a model’s latent size and its performance. Figure 4a visualizes the (**5**,**32**) model’s MCC scores as the latent size increases. As in the graph, the model shows the best performance when the latent size is 4 and then its performance fluctuates. Good performances were obtained when the latent sizes are small in the other two models as well; the (**5**,**64**) model achieves its best performance when the latent size is 3 (Figure 5a), and the (**7**,**64**) model achieves the MCC of about 0.7 which is close to its highest score at the latent size of 3 (Figure 6a).

We further inspect the model’s performance by the attack type. Figure 4b depicts the detection rate (TPR) per attack type as the latent size grows. The (**5**,**32**) model does particularly well on the detection of the DoS and Probe attacks: 0.892 at the latent size of 14 for DoS and 0.904 at the latent size of 7 for Probe. The performance for the other two attacks were low: 0.335 at the latent size of 9 for R2L and 0.646 at latent size of 12 for U2R.

>We see similar patterns for the (**5**,**64**) and (**7**,**64**) models. Figure 5a shows that the (**5**,**64**) model reaches its highest MCC performance with a small latent size of 3. This phenomenon is attributed to the dramatic increase in TPR of the R2L attacks, as shown in Figure 5b. The highest TPR was 0.892 for Probe and 0.901 for DoS when the latent size was 3. Both maintain a high stable performance after their peaks. On the other hand, for the U2R and R2L attacks the (**5**,**64**) model shows low TPR of 0.632 and 0.381, respectively. Additionally, there are large fluctuations in the R2L performance.

The (**7**,**64**) model also obtains a high MCC score at the small latent size of 3 and then fluctuates as the latent size grows (Figure 6a). Please note that the maximum number of the latent size in the (**7**,**64**) model is 15 not 31 as in the (**5**,**64**) model, due to another hidden layer of size 16 added to the encoder and the decoder. Like the (**5**,**64**) model, the DoS and Probe attack detection rates were high: 0.904 for DoS with the latent size of 9 and 0.901 for Probe with the latent size of 7. Those rates for the R2L and U2R attacks were low, 0.372 for R2L at the latent size of 9 and 0.653 for U2R at the latent size of 7. This provides evidence that an anomaly-based approach is not a relevant solution for detecting these types of attacks.

**Figure 7:**
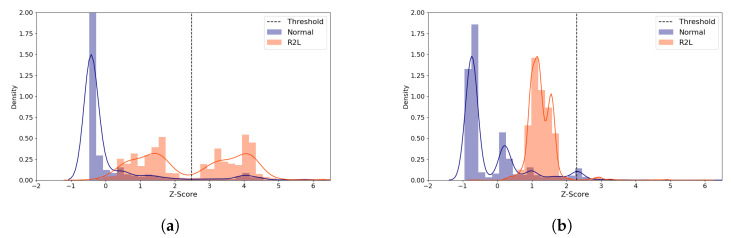
NSL-KDD Reconstruction Distance Density Plot (Normal and R2L attack data) comparing the best and the worst performance cases with the (**5**,**64**) model. Blue denotes normal data and orange denotes R2L attack data. A line depicts the kernel density estimated plot, and bar plot depicts the histogram. The dotted line indicates the threshold value, which divides the normal and the attack classes. The X-axis denotes Z-score. The Y-axis is density and limited to 2 for better visibility. (**a**) The best case (Latent size = 3, MCC = 0.712, Threshold = 2.488). (**b**) The worst case (Latent size = 1, MCC = 0.624, Threshold = 2.294).

We examine the role of the threshold value for ML-NIDS, by inspecting the reconstruction distance distribution of the R2L attacks of the (**5**,**64**) model. Despite its poor performance, this attack type was chosen because its TPR improved greatly as the latent size increased. We compare the average standardized reconstruction distance density plots of the R2L attacks using the (**5**,**64**) model with two distinct latent sizes (Figure 7). When the model performs best at the latent size of 3 as illustrated in Figure 7a, most normal samples are distributed below Z-score of 0. The attack samples are grouped into two parts, one part around Z-score of 1 and the other around Z-score of 4. The latter group is correctly classified as attack. On the other hand, when the latent size is 1 which produced the worst performance, Figure 7b shows that the distributions are skewed to the left side, mostly less than Z-score of 2, classifying the majority of the attack data as normal since its Z-score is less than the threshold. This shows that it is critical to choose the right latent size for building an autoencoder model for ML-NIDS.

### 5.3. Results: IoTID20 Data

This section describes our experiments using the IoTID20 dataset [49] which were collected in IoT network environments. We report the average performance of 10 runs for each of the three model configurations that is trained for 50 epochs with a batch size of 512. The average performance of each *Model Structure* at its best-performing latent size is presented in Table 8. The three *Model Structures* performed similarly, achieving an MCC score of up to 0.595, and this poor performance is due to its high FPR (>0.3). It is also noted that the accuracy is high (94%), despite the low MCC score.

Table 9 reports the best performance among the 10 runs for each model configuration. The TPR, FPR, accuracy, MCC, and F1-scores are presented for comparison with other works. The best F1-score of 0.974 was obtained for all the models. Table 10 displays the confusion matrix of the best-performing model for each configuration. The models make errors similar to those with NSL-KDD, predicting attacks as normal logs more often than predicting normal logs as attack.

**Figure 8:**
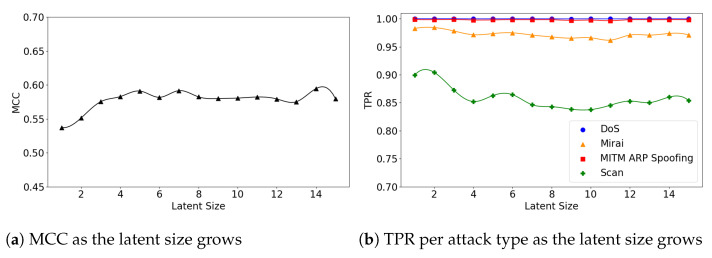
The results of evaluation using the IoTID20 dataset when the (**5**,**32**) model is employed.

We also explore the relationship between the model’s performance and its latent size. Figure 8a illustrates that the (**5**,**32**) model’s overall MCC gradually improves as the latent size grows, while TPR for the Scan attacks declines (Figure 8b). This is because the overall FPR also declines as the latent size increases.

**Figure 9:**
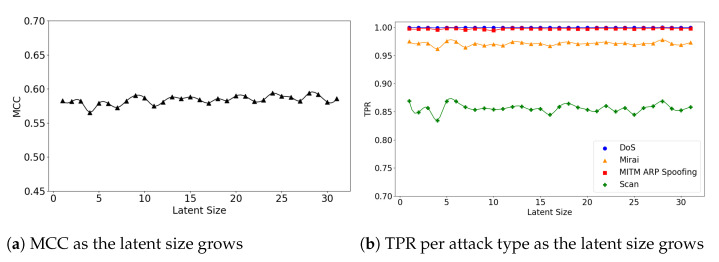
The results of evaluation using the IoTID20 dataset when the (**5**,**64**) model is employed.

**Figure 10:**
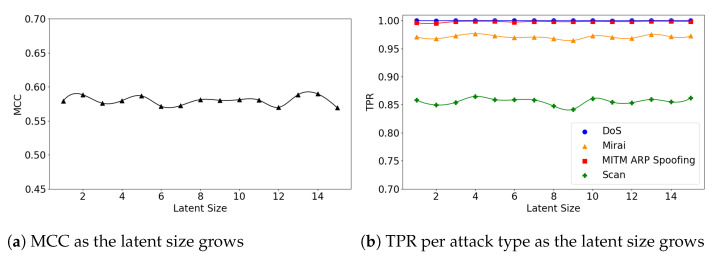
The results of evaluation using the IoTID20 dataset when the (**7**,**64**) model is employed.

The (**5**,**64**) and (**7**,**64**) models show little fluctuations in MCC, achieving near-best performance at small latent sizes. For example, the (**5**,**64**) model achieved MCC of 0.59 at the latent size of 9, which almost matches the best performance of 0.593 at the size of 28 (see Figure 9a). The (**7**,**64**) model also achieved MCC of 0.587 at the latent size of 5, and a near-best performance of 0.59 at the size of 14, as illustrated in Figure 10a. Across all three Model Structures, the detection rates of the DoS and MITM attacks were higher than those of the other attack types. In particular, the Scan attacks were the most difficult to detect (see Figure 9b and Figure 10b).

**Figure 11:**
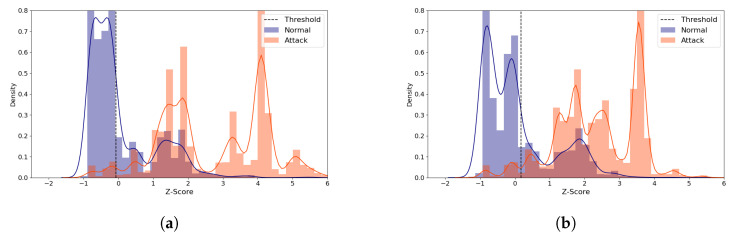
IoTID20 Reconstruction Distance Distribution Density Plot (Normal and attack data) comparing two similarly performing cases with the (**5**,**64**) model. Blue denotes the normal data and orange denotes the attack data. A line depicts the kernel density estimated plot, and bar plot depicts the histogram. The dotted line indicates the threshold value, which divides the normal and the attack classes. The X-axis denotes Z-score. The Y-axis is density and limited to 0.8 for better visibility. (**a**) The best case (Latent size = 28, MCC = 0.593, Threshold = 0.132). (**b**) A near-best case (Latent size = 9, MCC=0.59, Threshold = 0.132).

Figure 11 visualizes the reconstruction distance distributions of the normal and all the attack types with the (**5**,**64**) model for the latent sizes of 28 and 9, which produce the best and a near-best performance, respectively. Despite the significant difference in the latent size, the graphs in the figure show similar patterns; the normal samples are distributed between −1 and 3, and the most attack samples are distributed between 1 and 4. This suggests that using a small latent size can characterize the data well and efficiently.

### 5.4. Results: N-BaIoT Data

We performed experiments with the N-BaIoT data, separated into different sets based on the IoT device type—Danmini Doorbell, Philips Baby Monitor, SimpleHome Security Camera, Ecobee Thermostat, and Provision PT-737E. We report the average of 5 runs for each device type, where each run was trained for 50 epochs with a batch size of 1024. The average performance metrics of 5 runs of each model configuration for the selected IoT devices are available in Table 11.

As the table shows, the overall results are good as demonstrated by the accuracy greater than 99% and the MCC over 0.95. The best-performing model for the Danmini Doorbell device achieved the highest accuracy of 99.97% and an MCC of 0.996. This suggests that the attacks for the Danmini Doorbell are easily distinguishable via neural anomaly detection algorithms. The second-best performance was found for the Philips Baby Monitor, achieving an MCC of up to 0.989 and there was no significant difference in performance between the model configurations. It is also noted that the (**5**,**64**) model produced the best performance across all the device types.

**Figure 12:**
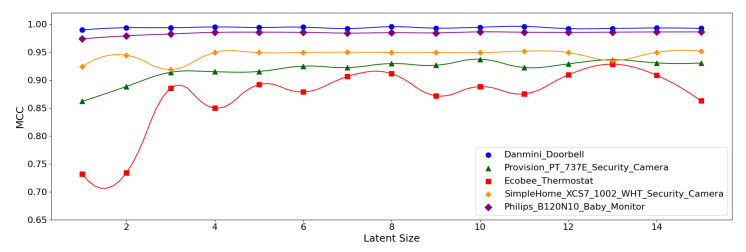
MCC per device for the N-BaIoT dataset when the (**5**,**32**) model is employed.

**Figure 13:**
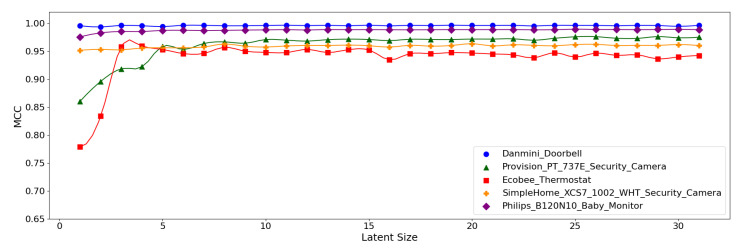
MCC per device for the N-BaIoT dataset when the (**5**,**64**) model is employed.

**Figure 14:**
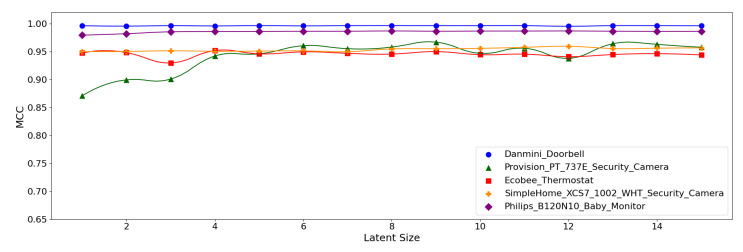
MCC per device for the N-BaIoT dataset when the (**7**,**64**) model is employed.

Figure 12, Figure 13 and Figure 14 display the MCC score of each *Model Structure* per device type as the latent size grows. Across all the model structures, Danmini Doorbell, Philips Baby Monitor, and SimpleHome Security Camera generally produce stable performance regardless of the latent size. We see a different trend for the Provision PT-737E and Ecobee Thermostat device types. The Provision PT-737E showed a significant improvement between the latent size of 1 and 4, across all the model structures. The Ecobee Thermostat with the (**5**,**32**) and (**5**,**64**) models show a dramatic improvement between the latent size of 1 and 3, followed by a relatively stable performance (Figure 12 and Figure 13); the (**5**,**64**) model achieved the highest MCC of 0.959 when the latent size was only 3 or 4. Such a significant change was not found when using the (**7**,**64**) model (Figure 14), since this model showed a stable performance regardless of the latent size factor.

**Figure 15:**
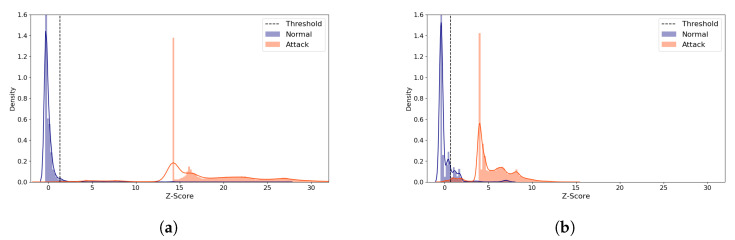
N-BaIoT Provision PT-737E Reconstruction Distance Distribution Density Plot (Normal and attack data) comparing the best and the worst performing cases when the (**5**,**64**) model was used. Blue denotes the normal data and orange denotes the attack data. A line depicts the kernel density estimated plot, and bar plot depicts the histogram. The dotted line indicates the threshold value, which divides the normal and the attack classes. The X-axis denotes Z-score. The Y-axis is density and limited to 1.6 for better visibility. (**a**) The best case (Latent Size = 29, MCC = 0.976, Threshold = 1.338). (**b**) The worst case (Latent Size = 1, MCC = 0.860, Threshold = 0.678).

Figure 15 displays the reconstruction distance distributions in Z-score of the normal and all the attack data when the (**5**,**64**) model was employed, comparing the distributions of the best and the worst performing cases. The distributions of the attack samples are very different, whereas the normal data tend to be concentrated within a narrow range of Z-score in both cases. In the best case (Figure 15a), the normal and the attack data rarely overlap. In addition, only a very small amount of normal data are greater than the threshold, which accounts for the high MCC score. On the other hand, Figure 15b shows the worst case where non-negligible number of normal samples are greater than the threshold, being falsely predicted as attack. In addition, some normal and attack data overlap between 1 and 2.

### 5.5. Discussions

#### 5.5.1. Model Structure and Performance

This section summarizes and discusses our primary findings. First, we look at the relationship between the model structure and performance in terms of the model capacity and its depth. In general, the (**5**,**64**) model—that contains 5 hidden layers and 64 neurons for the first hidden layer—produced the best or near-best performances across all the benchmark datasets. This indicates that the model capacity influences the IDS performance; models with large capacities tend to perform better. However, we did not find that the model depth (i.e., number of hidden layers) is associated with the performance. The model with less hidden layers (i.e., (**5**,**64**) model) outperformed the model with more hidden layers (i.e., (**7**,**64**) model), but the difference was not significant.

Second, we assess the IDS performance as the latent size varies. We observe that the overall intrusion detection performance tends to enhance as the latent dimension grows up to a certain point and decrease afterwards. This suggests that configuring the latent size can improve the performance greatly even when the model structure is fixed. The optimal latent size was diverse depending on the dataset, model capacity, and depth. For instance, the optimal latent size for the NSL-KDD data was 3 when using the (**5**,**64**) model. The optimal size for the IoTID20 data was 14 with the (**5**,**32**) model, but for N-BaIoT data, it was 28 when using the (**5**,**64**) model.

#### 5.5.2. Analysis of Reconstruction Errors and Threshold

Furthermore, we analyzed the distribution of the reconstruction errors in Z-score. The distributions of the normal and the attack samples are very different between the best and the worst cases. However, when the models produce identical performance, the samples are similarly distributed despite the difference in the latent size.

The distribution graphs also demonstrated that the threshold plays a critical role which determines the IDS performance in unsupervised learning methods (see Figure 7, Figure 11 and Figure 15). Previous unsupervised learning-based works [46,47] generally use *mean + standard deviation* as the threshold value, which is equal to Z-score of 1. In our observations, however, the best threshold differs based on the data, attack types, and the model configurations. For example, the best-performing threshold was about Z-score of 3 for the NSL-KDD and the Philips B120N/10 Baby Monitor datasets, but it was close to 0 for the IoTID20 dataset. Therefore, it is very challenging to find the threshold relevant to the dataset and the model configuration dynamically.

#### 5.5.3. Threats-to-Validity of Experimental Results

The following factors are the potential threats-to-validity of our experimental results: selection of model structures and latent dimensions to be compared and the types and properties of datasets. Since one of the major goals of our experiments is to elucidate the effect of model structure on IDS performance, selecting model structures to be compared may affect the validity of the experimental results. To see the impact of model capacity on IDS performance, we selected the (**5**,**32**) and (**5**,**64**) models that have the same number of hidden layers but only differ in the number of trainable parameters. To see the impact of the number of hidden layers, we compared the (**5**,**64**) and (**7**,**64**) models that have similar number of trainable parameters but different number of hidden layers. Similarly, the proper choice of latent dimensions to be compared is also crucial to analyze their impact on IDS performance, and we tested all possible latent dimensions for each model structure. The type of dataset is also an important factor that may affect the validity of the experimental results. To prevent experimental results from being biased to a specific dataset, we conducted experiments using three different types of datasets. Furthermore, we performed multiple runs of experiments for each dataset with random batch sampling of training data and random initialization of model weights to avoid biased experimental results.

## 6. Conclusions

Unsupervised deep-learning algorithms such as autoencoders have been actively studied for network intrusion detection since it can promptly detect the zero-day attack and alleviate the labor in labeling greatly [25,26]. Although various autoencoder models have shown to be effective in detecting intrusions, identifying the optimal model architecture to provide the best detection performance requires tremendous effort, and this hinders its practical application to NIDS. To address this engineering issue, we examine how the model architecture and the latent dimension affect the NIDS performance. This paper particularly focused on the model capacity, depth, and the size of the middle layer which represents the compressed, latent information of a given data.

We designed and carried out evaluation using a stacked autoencoder model with variations in model configurations. Empirical results on three datasets, NSL-KDD, IoTID20, and N-BaIoT, all collected from conventional and IoT networks, demonstrated that the model capacity and the latent size influenced the NIDS performance. Our simple stacked autoencoder models achieved the best F1-score of 0.895 with the NSL-KDD dataset, which is comparable to the best performance reported in the previous work [26]. In contrast, the performance does not depend on the model depth in our study. In addition, we noted that the simple autoencoders work surprisingly well on a particular IoT benchmark dataset, achieving the performance greater than 99% in accuracy and 0.96 in MCC, which is promising.

Our experimental results indicate that the model size (in terms of trainable parameters) and the size of the bottle neck layer have an impact on IDS performance. More specifically, a stacked autoencoder model tends to perform better and stable when its model size grows bigger. We also observe that the selection of the latent size can enhance the performance efficiently. From these findings, the researchers and practitioners working on NIDS can have benefited when exploiting their models to enhance the IDS performance without making major changes to the models. In addition, we discovered that a very small latent size can characterize the data very well in some cases, such as the IoTID20 dataset in our study. This may help build a lightweight IDS for IoT devices. For instance, Nykvist et al. [54] developed an efficient IDS that employed pattern matching algorithms for portable devices with limited processing power.

Our findings were drawn from the experiments using a limited set of configurations focusing on the model capacity, depth, and latent size. In particular, the impact of the depth is not fully confirmed in this study, and needs to be further examined using a larger autoencoder model. To build a large model, it is required to obtain datasets whose input feature vectors are large, since the input layer of the autoencoder is determined by the feature vector. Then, the sizes of the hidden layers of an autoencoder decrease down to the bottleneck layer. Future works can extend our work to cover other aspects of model design and an even wider variety of configurations and datasets. We will also look into methods to estimate the optimal latent size without scanning all possible ranges.

## Figures and Tables

**Table 1:** Count Statistics of the NSL-KDD dataset.

	Category	Training	Validation	Test
Benign	-	60,642	6701	9711
Attack	DoS	41,325	4602	7458
	U2R	50	2	200
	R2L	908	87	2754
	Probe	10,451	1205	2421

**Table 2:** Count Statistics of the IoTID20 dataset.

	Category	Train	Validation	Test
Benign	-	32,056	4001	4016
Attack	Mirai	332,569	41,744	41,364
	Scan	60,249	7409	7607
	DoS	47,519	5865	6007
	MITM	28,233	3560	3584

**Table 3:** Count Statistics for each device type of the N-BaIoT dataset.

Device	Label	Train	Validation	Test
Danmini Doorbell	Benign	39,675	4895	4978
	Attack	774,963	96,935	96,852
Ecobee Thermostat	Benign	10,528	1303	1282
	Attack	658,173	82,284	82,306
Provision PT-737E	Benign	49,753	6212	6189
	Attack	612,85	76,614	76,637
Philips B120N/10 Baby Monitor	Benign	140,069	17,574	17,579
	Attack	738,873	92,293	92,271
SimpleHome XCS7-1002-WHT	Benign	37,302	4620	4663
	Attack	655,143	81,685	81,643

**Table 4:** Model Structure Configurations.

Model Structure (Depth, Size)	Number of Neurons for Each Layer
(**5**,**32**)	*input – 32 – 16 – latent layer – 16 – 32 – output*
(**5**,**64**)	*input – 64 – 32 – latent layer – 32 – 64 – output*
(**7**,**64**)	*input – 64 – 32 – 16 – latent layer – 16 – 32 – 64 – output*

**Table 5:** Evaluation results with NSL-KDD. The best configuration from each model structure was chosen based on the MCC score. The reported metrics are averaged over 20 runs. The best MCC score is marked in bold.

Model Structure	Latent Size	Threshold	Accuracy	TPR	FPR	MCC	F1	AUC
(**5**,**32**)	4	2.846	0.840	0.757	0.051	0.705	0.842	0.960
(**5**,**64**)	3	2.488	0.848	0.782	0.065	**0.712**	0.853	0.960
(**7**,**64**)	9	2.840	0.847	0.777	0.061	0.711	0.852	0.959

**Table 6:** The best performance for each model configuration. The best F1-score is in bold.

Model Structure	Latent Size	Threshold	Accuracy	TPR	FPR	MCC	F1	AUC
(**5**,**32**)	4	2.120	0.887	0.851	0.066	0.778	**0.895**	0.961
(**5**,**64**)	3	2.090	0.885	0.859	0.081	0.771	0.894	0.961
(**7**,**64**)	9	2.340	0.882	0.826	0.045	0.774	0.888	0.971

**Table 7:** The confusion matrix of the best performing model for each configuration. Positive label indicates attack sample.

Model Structure	TP	FP	FN	TN
(**5**,**32**)	10,920	636	1913	9075
(**5**,**64**)	11,019	768	1814	8925
(**7**,**64**)	10,604	428	2229	9283

**Table 8:** Evaluation results with IoTID20 dataset. The best configuration from each model structure was chosen based on the MCC score. The reported metrics are averaged over 10 runs. The best MCC score is marked in bold.

Model Structure	Latent Size	Threshold	Accuracy	TPR	FPR	MCC	F1	AUC
(**5**,**32**)	14	0.041	0.945	0.963	0.313	**0.595**	0.971	0.912
(**5**,**64**)	28	−0.013	0.947	0.966	0.333	0.594	0.972	0.913
(**7**,**64**)	14	0.060	0.944	0.961	0.307	0.590	0.970	0.911

**Table 9:** The best performance for each model configuration. The best F1-score is in bold.

Model Structure	Latent Size	Threshold	Accuracy	TPR	FPR	MCC	F1	AUC
(**5**,**32**)	14	−0.350	0.951	0.971	0.329	0.614	**0.974**	0.909
(**5**,**64**)	28	−0.150	0.952	0.971	0.324	0.617	**0.974**	0.915
(**7**,**64**)	14	0.060	0.952	0.970	0.321	0.618	**0.974**	0.918

**Table 10:** The confusion matrix of the best performing model for each configuration. Positive label indicates attack sample.

Model Structure	TP	FP	FN	TN
(**5**,**32**)	55,683	1060	2841	2956
(**5**,**64**)	55,553	1017	2971	2999
(**7**,**64**)	52,480	815	6044	3201

**Table 11:** Evaluation results per device type with N-BaIoT Data. The best configuration from each model structure was chosen based on the MCC score. The reported metrics are averaged over 5 runs. The best MCC score of each device type is marked in bold.

(**a**) Danmini Doorbell								
**Model**	**Latent Size**	**Threshold**	**Accuracy**	**TPR**	**FPR**	**MCC**	**F1**	**AUC**
(**5**,**32**)	11	0.988	1.000	1.000	0.006	**0.996**	1.000	0.999
(**5**,**64**)	19	1.108	1.000	1.000	0.006	**0.996**	1.000	0.999
(**7**,**64**)	8	1.028	1.000	1.000	0.006	**0.996**	1.000	0.999
(**b**) Philips B120N / 10 Baby Monitor								
**Model**	**Latent Size**	**Threshold**	**Accuracy**	**TPR**	**FPR**	**MCC**	**F1**	**AUC**
(**5**,**32**)	10	3.130	0.996	0.998	0.013	0.986	0.998	0.999
(**5**,**64**)	25	3.270	0.997	0.999	0.011	**0.989**	0.998	0.999
(**7**,**64**)	8	3.344	0.996	0.998	0.012	0.987	0.998	0.999
(**c**) SimpleHome XCS7-1002-WHT Security Camera								
**Model**	**Latent Size**	**Threshold**	**Accuracy**	**TPR**	**FPR**	**MCC**	**F1**	**AUC**
(**5**,**32**)	11	0.438	0.995	0.999	0.074	0.952	0.997	0.990
(**5**,**64**)	20	1.050	0.996	0.999	0.051	**0.963**	0.998	0.998
(**7**,**64**)	12	0.846	0.996	0.999	0.058	0.959	0.998	0.996
(**d**) Provision PT-737E								
**Model**	**Latent Size**	**Threshold**	**Accuracy**	**TPR**	**FPR**	**MCC**	**F1**	**AUC**
(**5**,**32**)	10	0.854	0.991	0.994	0.050	0.937	0.995	0.992
(**5**,**64**)	29	1.338	0.997	0.999	0.027	**0.976**	0.998	0.999
(**7**,**64**)	9	1.102	0.995	0.998	0.040	0.967	0.998	0.999
(**e**) Ecobee Thermostat								
**Model**	**Latent Size**	**Threshold**	**Accuracy**	**TPR**	**FPR**	**MCC**	**F1**	**AUC**
(**5**,**32**)	13	0.802	0.998	0.999	0.067	0.928	0.999	0.997
(**5**,**64**)	4	0.408	0.999	1.000	0.064	**0.959**	0.999	0.999
(**7**,**64**)	4	0.366	0.999	1.000	0.079	0.952	0.999	0.999

## Data Availability

The datasets used in this study are publicly available for academic research purposes. NSL-KDD https://www.unb.ca/cic/datasets/nsl.html (accessed on 21 June 2021). IoTID20 https://sites.google.com/view/iot-network-intrusion-dataset/home (accessed on 21 June 2021). N-BaIoT https://archive.ics.uci.edu/ml/datasets/detection_of_IoT_botnet_attacks_N_BaIoT (accessed on 21 June 2021).
